# Insights from the Pre-Molecular Era in Advanced Endometrial Cancer: Benchmarking Prognostic Indicators in High-Risk Tumours

**DOI:** 10.3390/jcm14248726

**Published:** 2025-12-09

**Authors:** Jacopo Conforti, Sabina Ioana Nistor, Negin Sadeghi, Andreas Zouridis, Ammara Kashif, Ahmed Darwish, Sarah Louise Smyth, Alisha Sattar, Susan Addley, Christina Pappa, Stephen Damato, Mostafa Abdalla, Sean Kehoe, Andrea Giannini, Federico Ferrari, Hooman Soleymani Majd

**Affiliations:** 1 Department of Gynaecology Oncology, Churchill Hospital, Oxford University Hospitals, National Health Service (NHS) Foundation Trust, Oxford OX3 7LE, UK; 2 Department of Clinical and Experimental Sciences, University of Brescia, 25136 Brescia, Italy; 3 Nuffield Department of Women’s and Reproductive Health, University of Oxford, Oxford OX2 6GG, UKahmed.darwish@ouh.nhs.uk (A.D.);; 4 Wexham Park Hospital, Frimley Health NHS Foundation Trust, Slough SL2 4HL, UK; 5 University Hospital of Derby and Burton NHS Foundation Trust, Derby DE2 3NE, UK; 6 Department of Histopathology, Oxford University Hospitals NHS Foundation Trust, Oxford OX3 9DU, UK; 7 Guy’s and St Thomas’ NHS Foundation Trust, London SE1 9RT, UK; 8 Unit of Gynecology, Department of Surgical and Medical Sciences and Translational Medicine, Sant’Andrea Hospital, Sapienza University of Rome, 00185 Rome, Italy; andrea.giannini@uniroma1.it

**Keywords:** endometrial cancer, advance stage, endometrioid endometrial cancer, carcinosarcoma, serous carcinoma, clear cell carcinoma, molecular mutation

## Abstract

**Background/Objectives**: Although the binarism between type I and II endometrial cancer was dismissed and substituted with molecular classification, histopathological features remain of paramount importance. Hence, analysing survival outcomes according to histological type, our aim is to clarify whether the morphological features of the tumour retain prognostic relevance in the context of advanced disease. **Methods**: This is a retrospective analysis led within the Thames Valley Cancer Alliance Network. **Results**: We include 148 FIGO 2009 stage III–IV patients affected by endometrioid endometrial cancer (EEC) G1, G2, and G3, carcinosarcoma (CS), serous carcinoma (SC), and clear cell carcinoma (CCC) of the uterus. Five year overall survival (OS) is distinct among the histological groups (*p*-value < 0.001), being 73.3% for G2 endometrioid, 49.2% for G3 endometrioid, 8.3% for CS, and 28.4% for SC. The divergence was marked also for 5 year progression-free survival (PFS) (*p*-value < 0.001) as follows: for G2 endometrioid, was 76.4%; for G3 endometrioid, 52.7%; and for carcinosarcoma, 5.9%. PFS after 18 months for serous carcinoma was 5.7%. The multivariate analysis found G3 endometrioid (HR 2.91, 95% CI 1.20–7.11, *p*-value 0.018), carcinosarcoma (HR 12.15, 95% CI 5.07–29.11, *p*-value < 0.001), and serous carcinoma (HR 4.84, 95% CI 2.16–10.83, *p*-value < 0.001) as independent predictors of poor survival, as well as cervical invasion (HR 1.83, 95% CI 1.10–3.05, *p*-value 0.020) as the only histopathological feature confirmed. Regarding progression-free only carcinosarcoma (HR 14.91, 95% CI 5.28–41.11) and serous carcinoma (HR 17.68, 95% CI 6.41–48.75) were associated with an increased risk of recurrence. **Conclusions:** Our findings testify that, beyond the disease stage, histological subtype remains a major determinant of survival outcome. Cervical involvement is associated with a more aggressive disease, possibly correlated to death beyond relapse. Prospective trials involving advanced stage endometrial cancer, stratified by histological subtype and integrated with the molecular classification, are required.

## 1. Introduction

According to the World Cancer Research Fund [1], endometrial cancer (EC) ranks as the sixth most common malignancy among women, with more than four hundred thousand new cases diagnosed in 2022. The 2023 guidelines of the International Federation of Gynaecology and Obstetrics (FIGO) [2] classified EC into the following distinct subtypes: low-grade (G1 and G2) endometrioid endometrial carcinoma (EEC) and high-grade tumours, including high-grade (G3) EEC, serous carcinoma (SC), carcinosarcoma (CS), clear cell carcinoma (CCC), mixed carcinoma, undifferentiated carcinoma, and unusual types. Moreover, the traditional dichotomy between type I and II [3] endometrial carcinoma has been abandoned, replaced by the molecular classification introduced by The Genome Cancer Atlas (TGCA) project [4]. Molecular features were already incorporated in the FIGO 2023 [2] staging system, allowing for both disease stage upgrades and downgrades. Nevertheless, the molecular profiling is not solely used for the classification, as the RAINBO (Refining Adjuvant Treatment in Endometrial Cancer Based on Molecular Features) clinical trial programme is currently exploring how to tailor adjuvant therapies to specific molecular subgroups.

Although the binarism between type I and II was dismissed, histopathological features remain of paramount importance. EECs are graded as G1, G2, and G3 based on architecture, texture, and cytological criteria [5]; furthermore, G1 and G2 EECs are the most common subtypes and are associated with well-established risk factors such as obesity, age, family history, late menopause, prolonged oestrogen therapy, and ethnicity. CS, though rare, has a poorer prognosis and is usually diagnosed in an advanced stage, but also early stages have a negative prognosis [6]. Similarly, SC is not a common disease but often presents with metastatic presentation and poor outcome [7], while CCC is even more rare, with an aggressive behaviour and a marked chemoresistant [8].

In this work we focused only on advanced-stage endometrial cancer (FIGO 2009 Stage III–IV) to verify whether the histology of the tumour changes the prognosis or not and to assess the impact of adjuvant therapy on clinical outcomes. Different histological categories exhibit distinct biological behaviour and treatment responses; nevertheless, their prognostic differences in advanced disease are not fully understood. Hence, analysing survival outcomes according to histological type, our aim was to clarify whether the morphological features of the tumour retain prognostic relevance in the context of advanced disease. We believe that understanding these bonds might supply practical tools to be integrated with the molecular classification.

## 2. Materials and Methods

This is a retrospective analysis led within the Thames Valley Cancer Alliance Network, a catchment area of 2.3 million people. Data collection spanned a 10 year period, from January 2010 to December 2020, and was carried out at Churchill Hospital, Oxford University Hospital Foundation Trust. This manuscript is part of a broader research project with the aim to analyse clinical and histopathological patterns of recurrence and survival of endometrial cancers.

Patients were recruited according to the listed inclusion criteria: age over 18 years of age, the diagnosis of endometrial cancer, and suitability for surgical management. Women under 18 years, presence of synchronous tumours, or the absence of clinical and histopathological reports or follow-up data were excluded from the analysis. For the purpose of this study, only stages III and IV, according to The International Federation of Gynaecology and Obstetrics (FIGO) 2009, were included.

Data acquisition encompassed medical and surgical history, surgical and histopathological reports, and follow-up information. These variables were subsequently used for the statistical analysis. Histopathological patterns included in the current study were EEC G1, G2, and G3, as well as SC, CS, and CC. Mixed tumours were excluded. All cases were approached at the multidisciplinary team discussion (MDT), both before and after the surgery, to define the operative approach and the feasibility of adjuvant treatment, according to national guidelines.

The Oxford University Hospitals Trust requirements registered the service evaluation protocol (registration number 5832). The conception of the study, data analysis, interpretation, manuscript drafting, and subsequent revisions were carried out in accordance with the principles of the Declaration of Helsinki, the recommendations of the Committee on Publication Ethics, and the reporting of studies conducted using observational routinely collected health data (RECORD) guidelines, endorsed by the Enhancing the Quality and Transparency of Health Research Network (EQUATOR). All information was pseudoanonymised, due to the observational design of the research, and no personal element that might allow patient identification was collected. Every participant was adequately informed about the procedures and provided written consent for the use of their clinical data for scientific purposes.

The statistical analysis was performed using IBM©SPSS Statistics 22.0. Continuous variables were analysed using Student’s *t*-test, while survival outcomes were estimated using Kaplan–Meier and compared with the log-rank test. Risk factors that were chosen for the inquiry were assessed with the univariate and multivariate Cox proportional hazard models. A *p*-value inferior to 0.05 was considered statistically significant. Variables included in the multivariate analysis were those that resulted in statistical significance from previous works of this group [9,10,11].

## 3. Results

### 3.1. Descriptive Analysis

One hundred forty-eight (148) patients with FIGO stage III–IV were surgically treated at Churchill Hospital, and the average age at surgery was 68.8 years (SD ± 10.4 years). Histopathological reports confirmed EEC G1 in 14 patients (9.5%), G2 in 31 (20.9%), and G3 in 24 (16.2%), whereas SC and CS were diagnosed in 42 (28.4%) and 32 (21.6%) patients, respectively. At least, only 5 cases of CC (3.4%) were operated on.

Laparoscopic surgery represented the predominant approach, used in 107 cases (72.3%), while the open approach was indicated only for 37 women (25.0%). Surgical lymph node assessment was undertaken in just 79 cases (53.4%), including 16 (10.8%) with groin sampling and 63 (41.2%) who underwent systematic pelvic and/or paraortic dissection. Unfortunately, 4 cases were omitted from the descriptive surgical analysis due to missing operative notes data. Demographic and surgical information, organised by histotype, is displayed in Table 1.

Histopathological description (Table 2) revealed a vast local disease spread across most cases. Deep myometrial invasion (≥50%) and lymphovascular space invasion (LVSI) were the most common features; both occurred in 118 patients (79.7%). Cervical stroma invasion was detected in 67 cases (45.3%), uterine serosal invasion in 60 patients (40.5%), and adnexa and parametrial involvement in 52 specimens (35.1%) each.

The distribution of the FIGO 2009 classification for every histopathological variant is represented in Table 3. Stage IIIA was the most prevalent, recognised in 52 patients (35.1%). Noteworthily, stage IIIC1 was more frequent than stage IIIB (24.3% vs. 20.9%), while stages IIIC2 and IVA were rare, viewed in 8 (5.4%) and 1 (0.7%) case, respectively. Stage IVB was observed in 20 patients (13.5%).

Adjuvant treatment (chemotherapy, radiotherapy, brachytherapy or a combination) was prescribed in 93 cases out of 148 (62.8%), after MDT. Among these, 5 patients (5.4%) with G1 EEC received adjuvant therapy, as well as 17 women (18.3%) with G2 EEC and 16 (17.2%) with G3 EEC. Twenty-nine patients (31.2%) with SC and 26 (28.0%) with CS took in adjuvant therapy. Unfortunately, follow up data for patients with CC were not available.

Recurrences were detected in 60 women (40.5%), with a median recurrence time of 10 months (IQR 25–75% 4–21 months). In 18 (30.0%) cases the metastases were in a single area as vaginal vault, omentum, lymph nodes and lungs, whereas 42 (70%) patients presented multisite relapses. Interesting, all the recurrences for G1, G2 and G3 EEC were locals or at the lungs, while CS and SC show a more widespread pattern for relapses, ranging from locals and pelvic lymph glands, but also involving bladder, bowel, liver, omentum, lungs, bones, kidneys, adrenal glands and paracardic glands. The distribution of the relpse of the disease was as follows: 6 (10.0%), 9 (15.0%) and 8 (13.3%) for G1, G2 and G3 EEC, while 19 (31.7%) and 18 (30.0%) in CS and SC.

### 3.2. Survival and Univariate Analysis

Due to the small number of events, limited sample size, and missing data, G1 EEC and CC were not included in the survival analysis.

**Figure 1:**
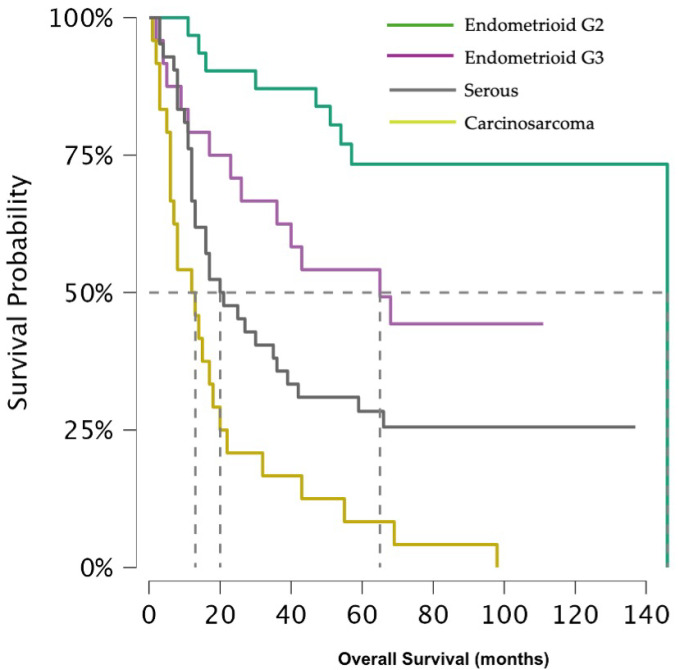
Shows overall survival across histological subtypes.

The difference between 5 year overall survival (OS) is distinct among the histological groups (*p*-value < 0.001) (Figure 1), being 73.3% (95% CI 59.1–91.1%) for G2 EEC and 49.2% (95% CI 32.6–74.4%) for G3 EEC, while a massive decline was observed for CS and SC, with a 5 year OS of 8.3% (95% CI 2.2–31.4%) and 28.4% (95% CI 17.5–46.0%), respectively.

**Figure 2:**
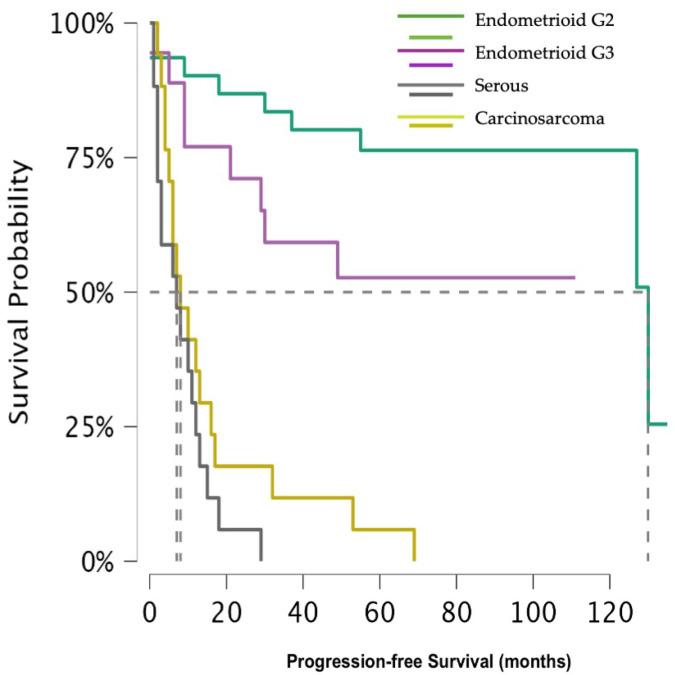
Shows progression-free survival across histological subtypes.

In relation to the 5 year risk of recurrence, the divergence was keen as well (*p*-value < 0.001) (Figure 2). The 5 year progression-free survival (PFS) for G2 EEC was 76.4% (95% CI 62.4–93.4%), for G3 EEC 52.7% (95% CI 33.4–83.1%), and 5.9% (95% CI 0.9–39.4%) for CS. Due to the small number of events occurring after 18 months, the 5 year PFS for SC could not be calculated, but at that time was 5.7% (95% CI 0.9–39.4%).

The Cox univariate for OS analysis including only the histotype revealed a worse outcome for G3 EC (*p*-value 0.028), CS (*p*-value < 0.001), and SC (*p*-value < 0.001) compared to G2 EEC, with an hazard ratio (HR) of 2.68 (95% CI 1.11–6.48), 10.52 (95% CI 4.67–23.71) and 4.89 (95% CI 2.23–10.69) for G3 EEC, CS and SC, accordingly. For PFS, G3 EEC had a HR of 2.42 (95% CI 0.87–6.68) than G2 EEC, however this result was not statistically significant (*p*-value 0.88). In contrast, both CS and SC were associated with a markedly higher risk of recurrence (*p*-value < 0.001 for both), with a HR notably exceeding those observed for OS. Indeed, CS showed a HR of 11.56 (95% CI 4.64–28.79), while SC of 18.23 (95% CI 6.96–47.73).

A subgroup analysis based on adjuvant treatment revealed a significant difference only in G2 EEC, where the log-rank test indicated a reduce risk of recurrence (*p* value = 0.039); however, this result was not confirmed in the Cox univariate analysis (Table 4).

### 3.3. Multivariate Analysis

In the multivariate analysis we included all the variables that resulted statistically significant in our previous work that treated the single histologies (myometrial invasion equal or more than 50%, LVSI, parametrium, cervical stroma and serosal involvement), assessed across the five histological subgroups included in the present project.

The Cox regression for the OS revealed CS (HR 12.15, 95% CI 5.07–29.11, *p*-value < 0.001), SC (HR 4.84, 95% CI 2.16–10.83, *p*-value < 0.001), G3 EEC (HR 2.91, 95% CI 1.20–7.11, *p*-value 0.018) and cervical involvement (HR 1.83, 95% CI 1.10–3.05, *p*-value 0.020) as independent predictors of worse survival. Serosal invasion approached statistical significance (*p*-value 0.058), showing a trend toward an increased risk of death with a HR 1.62 (95% CI 0.98–2.67). All the other factors included were not statistically significant.

With regard to PFS, CS and SC remained strongly associated with an increased risk of recurrence (*p*-value < 0.001), with a HR of 14.91 (95% CI 5.28–41.11) and 17.68 (95% CI 6.41–48.75), respectively. In this case, G3 EEC was close to significance (*p*-value 0.055) with a HR for recurrence of 2.75 (95% CI 0.97–7.75).

## 4. Discussion

Among gynaecological malignancies, uterine cancer has benefited the most from the advent of molecular classification. The new FIGO 2023 staging system not only introduced new stages according to POLE and p53 mutation, but also four risk classes related to histotype and grade, mutations, and FIGO stage [2].

In the present study, we specifically evaluated patients with advanced stage disease (FIGO 2009 stage III–IV) to determine whether histology continues to influence prognosis in this subset. Our findings testify that, beyond the disease stage, histological subtype remains a major determinant of survival outcome. Women affected by G2 and G3 EEC exhibited substantially higher 5 year OS rates (73.3% and 49.2%) compared with those with CS and SC (5 year OS 8.2% and 28.4%). A similar pattern was observed for progression-free survival: 5 year PFS for CS was 5.9%, and at 18 months was 5.7% for SC. These results were also confirmed in the multivariate analysis, which identified cervical involvement as an independent predictor of worse OS, though not of increase recurrence risk. Although adjuvant medical therapy was administered, the small numbers within each specific treatment subgroup rendered further stratified survival analyses statistically underpowered and potentially misleading. For this reason, regimen-specific survival comparisons were not undertaken, as they would not have provided methodologically robust or clinically reliable conclusions.

Our survival results confirm what was previously reported in the literature. A retrospective study [12] including more the two hundred thousand patients comparing survival outcomes across endometrial cancer stages revealed a 5 year OS for stage III G2 EEC of 68.9%, for G3 EEC of 49.6% and for SC of 35.7%. Although, this research did not assess the 5 year PFS nor included CS, its findings corroborate the prognostic hierarchy we observed. Mhawech-Fauceglia et al. [13] described a higher recurrence and mortality rate for SC compared to EEC when considering stage II–III–IV, but when restricting the analysis only to advanced disease the difference did not reach the statistical significance.

This research confirms what our group has previously demonstrated about the correlation between cervical involvement and poorer overall survival [9,10]. Conversely, including different histologies for advanced stage EC, the cervical invasion is not correlated to a worsening of 5 year PFS. Noteworthily, cervical involvement did not affect survival or recurrence in early stage disease when considering CS, CC, and G3 EEC [14]. Specifically, in another research the multivariate analysis failed to confirm the association between cervical stroma involvement and distant metastasis when considering stage I-II endometrioid endometrial cancer [15]. While cervical stroma invasion deteriorates the overall survival in our cohort, this does not happen for the PFS. A possible explanation could be that the cervical involvement is associated with a more aggressive disease, correlated to death beyond relapse. Therefore, women could pass away due to high tumour burden and comorbidities, before manifest a recurrence. Lately, a disease that spread to the cervix could also be less sensitive to adjuvant treatment.

Myometrial invasion has long been recognised as an adverse prognostic factor for both OS and PFS [16]. Zouridis [9] and Smyth et al. [10] confirmed these features in their works on G3 EEC and CS, respectively, as well as Wang et al. [17] did in their work of high grade endometrioid endometrial cancer. Furthermore, the influence of myometrial invasion on survival outcome was also confirmed in other studies including CS [18]. On the other hand, other authors [19] did not confirm this data on G3 EEC and CS in a similar settings.

Parametrial involvement was found to be predictive of higher mortality in G2 EEC [11]. Barquet-Munoz et al. [20] analysed factors associated with parametrial involvement and the effect of lateral invasion on survival considering a population from stage I to IV with endometrial carcinoma, founding the negative effect on OS and PFS. Conversely, when considering only stage III–IV and subtypes like G2 and G3 EEC, CS and SC we did not find any correlation between parametrial invasion and worsening of any outcomes.

Special attention should be given to LVSI, whose importance was emphasised in the latest FIGO 2023 classification [2]. Wang and Zhu [17,19] demonstrated the prognostic role for recurrence and survival of LVSI only in the univariate analysis, while adjusting for other factors they were not be able to confirm the result. This is consistent with our research and those of other investigators [21]. In contrast, a previous retrospective [22] restricted to FIGO 2009 stage III–IV cancer reported LVSI as an independent predictors of poor survival, as well as myometrial invasion, though CS and SC were excluded from that analysis.

Our study is limited by the retrospective design and the lack of molecular classification, which were not yet integrated in the clinical practice during the study period. Molecular classification has been shown to upgrade mainly stage I–II [23], without this substantive effect on advanced stages. However, molecular classification can expand the adjuvant treatment selection with a target therapy, therefore this is an embedded weakness of this study. Nevertheless, the centralization of these women ensured consistent management and reduced surgical variability. Moreover, few studies have focused exclusively on advanced stages, as most include early disease; thus, our cohort size and stage-specific focus represent additional strengths. Overall, our findings may serve as a valuable complement to molecular classification, emphasising that even in the era of genomic classification, tumour stage and histological subtype continue to play a fundamental prognostic role, as confirmed in another recent work [24]. Furthermore, some of our results are aligned with the current literature, however the retrospective nature of both our study and previous reports introduces inherent biases that might explain the inconsistencies reported across studies.

## 5. Conclusions

Our study demonstrates that the prognosis of advanced endometrial cancer is not uniform across histological subtypes. Carcinosarcoma and serous endometrial cancer exhibit the poorest survival outcomes, whereas most conventional prognostic features were not confirmed, apart from cervical invasion, which remained independently associated with reduced overall survival.

This is to say that prospective trials involving advanced stage endometrial cancer, stratified by histological subtype and integrated with the molecular classification, are required to better define prognostic determinants and to refine individualised treatment strategies.

## Figures and Tables

**Table 1:** Summary of histotype distribution and surgical reports.

		EEC G1	EEC G2	EEC G3	Serous Carcinoma	Carcinosarcoma	Clear Cell
		(%)	(%)	(%)	(%)	(%)	(%)
	Number	14 (9.5)	31 (20.9)	24 (16.2)	42 (28.4)	32 (21.6)	5 (3.4)
General							
	Mean age (years)	58.1	70.2	69.9	69.2	71.3	66.0
	Laparoscopy	11 (7.4)	26 (17.6)	17 (12.2)	24 (16.2)	28 (18.9)	1 (0.7)
	Laparotomy	3 (2.0)	2 (1.4)	7 (4.7)	17 (11.5)	4 (2.7)	4 (2.7)
	Missing data	0	3	0	1	0	0
Surgery							
	Lymph node	1 (0.7)	2 (1.4)	5 (3.4)	4 (2.7)	3 (2.0)	1 (0.7)
	sampling						
	Systematic lymph	1 (0.7)	6 (4.1)	12 (8.1)	20 (13.5)	20 (13.5)	4 (2.7)
	node dissection						

Legend: EEC—Endometrioid endometrial cancer.

**Table 2:** Local histopathological features.

	EEC G1	EEC G2	EEC G3	Serous Carcinoma	Carcinosarcoma	Clear Cell
	(%)	(%)	(%)	(%)	(%)	(%)
Myometrial invasion > 50%	10 (6.8)	23 (15.5)	20 (13.5)	31 (20.9)	29 (19.6)	5 (3.4)
Cervical invasion	4 (2.7)	17 (11.5)	10 (6.8)	21 (14.2)	14 (9.5)	1 (0.7)
Adnexa invasion	5 (3.4)	7 (4.7)	6 (4.1)	23 (15.5)	10 (6.8)	1 (0.7)
Serosal invasion	4 (2.7)	8 (5.4)	10 (6.8)	19 (12.8)	18 (12.2)	1 (0.7)
Parametrial invasion	1 (0.7)	14 (9.5)	9 (6.1)	15 (10.1)	12 (8.1)	1 (0.7)
Lymphovascular	6 (4.1)	24 (16.2)	20 (13.5)	34 (23.0)	29 (19.6)	5 (3.4)
space invasion						

Legend: EEC—Endometrioid endometrial cancer.

**Table 3:** FIGO 2009 classification for each histologycal subtype included in the research.

	EEC G1	EEC G2	EEC G3	Serous Carcinoma	Carcinosarcoma	Clear Cell
	(%)	(%)	(%)	(%)	(%)	(%)
IIIA	10 (6.8)	13 (8.8)	7 (4.7)	12 (8.1)	10 (6.8)	0
IIIB	1 (0.7)	14 (9.5)	5 (3.4)	7 (4.7)	2 (1.4)	2 (1.4)
IIIC1	1 (0.7)	3 (2.0)	7 (4.7)	12 (8.1)	10 (6.8)	3 (2.0)
IIIC2	0	1 (0.7)	3 (2.0)	2 (1.4)	2 (1.4)	0
IVA	0	0	0	0	1 (0.7)	0
IVB	2 (1.4)	0	2 (1.4)	9 (6.1)	7 (4.7)	0

Legend: EEC—Endometrioid endometrial cancer.

**Table 4:** Subgroup analysis comparing patients who received adjuvant treatment and who did not.

	All Histotypes		EEC G2		EEC G3		Carcinosarcoma		Serous Carcinoma	
	OS	PFS	OS	PFS	OS	PFS	OS	PFS	OS	PFS
Log-rank test										
*p*-value	0.107	0.135	0.340	0.039	0.465	0.568	0.827	0.288	0.134	0.320
Cox univariate analysis										
*p*-value	0.113	0.138	0.353	0.060	0.471	0.671	0.847	0.308	0.149	0.303
95% CI	0.352–1.118	0.288–1.188	0.081–2.448	0.043–1.066	0.119–2.675	0.075–5.282	0.293–2.739	0.367–23.889	0.216–1.262	0.151–1.801

Legend: EEC—Endometrioid endometrial cancer.

## Data Availability

Data are available under reasonable request and after the approval of the corresponding author in order to protect the privacy of our patients.

## Abbreviations

The following abbreviations are used in this manuscript:

EC	Endometrial cancer
FIGO	The International Federation of Gynaecologist and Obstetrics
G1/G2	Low-grade
EEC	Endometrioid endometrial carcinoma
G3	High-grade
SC	Serous carcinoma
CS	Carcinosarcoma
CCC	Clear Cell carcinoma
TGCA	The Genome Cancer Atlas
RAINBO	Refining Adjuvant Treatment in Endometrial Cancer Based on Molecular Features
MDT	Multidisciplinary team
RECORD	Reporting od studies Conducted using Observational Routinely collected Health Data
EQUATOR	Enhancing the Quality and Transparency of Health Research Network
LVSI	Lymphovascular space invasion
OS	Overall survival
PFS	Progression-free survival
HR	Hazard ratio
CI	Confidence interval
